# Illicit drug use while admitted to hospital: Patient and health care provider perspectives

**DOI:** 10.1371/journal.pone.0229713

**Published:** 2020-03-05

**Authors:** Carol Strike, Samantha Robinson, Adrian Guta, Darrell H. Tan, Bill O'Leary, Curtis Cooper, Ross Upshur, Soo Chan Carusone

**Affiliations:** 1 Dalla Lana School of Public Health, University of Toronto, Toronto, Canada; 2 School of Social Work, University of Windsor, Windsor, Canada; 3 St. Michael's Hospital, Toronto, ON, Canada; 4 Casey House, Toronto, ON, Canada; 5 Division of Infectious Diseases, University of Ottawa, Ottawa, Canada; 6 McMaster University, Hamilton, ON, Canada; University of California San Diego, UNITED STATES

## Abstract

**Background:**

Across North America, the opioid overdose epidemic is leading to increasing hospitalizations of people who use drugs (PWUD). However, hospitals are ill-prepared to meet the needs of PWUD. We focus on illicit drug use while admitted to hospital and how PWUD and health care providers describe, respond, and attempt to manage its use.

**Methods and findings:**

Using varied purposive methods in Toronto and Ottawa, we recruited n = 24 PWUD (who self-reported that they were living with HIV and/or HCV infection; currently or had previously used drugs or alcohol in ways that were harmful; had a hospital admission in the past five years) and n = 26 health care providers (who were: currently working in an academic hospital as a physician, nurse, social worker or other allied health professional; and 2) providing care to this patient group). All n = 50 participants completed a short, socio-demographic questionnaire and an audio-recorded semi-structured interview about receiving or providing acute care in a hospital between 04/2014 and 05/2015. Patient participants received $25 CAD and return transit fare; provider participants received a $50 CAD gift card for a bookseller. All participants provided informed consent. Audio-recordings were transcribed verbatim, corrected, and uploaded to NVivo 10. Using the seven-step framework method, transcripts were coded line-by-line and managed using NVvivo. An analytic framework was created by grouping and mapping the codes. Preliminary analyses were presented to advisory group members for comment and used to refine the interpretation. Questionnaire data were managed using SPSS version 22.0 and descriptive statistics were used to describe the participants. Many but not all patient participants spoke about using psycho-active substances not prescribed to them during a hospital admission. Attempts to avoid negative experiences (e.g., withdrawal, boredom, sadness, loneliness and/or untreated pain) were cited as reasons for illicit drug use. Most tried to conceal their illicit drug use from health care providers. Patients described how their self-reported level of pain was not always believed, tolerance to opioids was ignored, and requests for higher doses of pain medications denied. Some health care providers were unaware of on-site illicit drug use; others acknowledged it occurred. Few could identify a hospital policy specific to illicit drug use and most used their personal beliefs to guide their responses to it (e.g., ignore it, increase surveillance of patients, reprimands, loss of privileges/medications, threats of immediate discharge should it continue, and substitution dosing of medication).

**Conclusions:**

Providers highlighted gaps in institutional guidance for how they ought to appropriately respond to in-hospital substance use. Patients attempted to conceal illicit drug use in environments with no institutional policies about such use, leading to varied responses that were inconsistent with the principles of patient centred care and reflected personal beliefs about illicit drug use. There are increasing calls for implementation of harm reduction approaches and interventions in hospitals but uptake has been slow. Our study contributes to this emerging body of literature and highlights areas for future research, the development of interventions, and changes to policy and practice.

## Introduction

Across the world, rates of opioid related overdose vary with the United States and Canada reporting rates four times the global average and Australia and New Zealand combined reporting twice the rate of the global average [1–4]. The magnitude of this crisis is reflected in a decline in overall life expectancy in the United States [5] and some regions of Canada [6, 7]. As this epidemic has evolved, hospitalizations of people for overdose and injection drug use-related problems have increased as have calls for innovative models of care [8, 9]. While hospitalizations can be unpleasant for the average person, they are especially challenging for people who use drugs (PWUD) who commonly have histories of social, medical, and structural complexity, as well as stigma and resulting trauma [10]. Priest and McCarty contend that acute care for PWUD is needed, possible and effective [11]. However, the extant literature shows that few hospitals provide optimal care for PWUD and, with a few exceptions at specific hospitals, policies regarding the inpatient care of any medical problem among PWUD are lacking despite calls for such guidelines [12–15].

Mutual distrust between health care providers and PWUD has been documented to influence therapeutic interactions in hospitals and impacts the quality of care delivered [16, 17]. In these settings, PWUD are often perceived to be problematic and drug-seeking by health care providers [13, 14, 18]. Correspondingly, PWUD often perceive being stigmatized and report that the care they receive is substandard compared with other patients [16].

Hospital settings have been characterized as risk environments [19, 20] wherein contextual factors (e.g., formal and informal policies, discourses, and discrimination) external to the individual produce and shape harms. McNeil and colleagues applied this approach to examine the experiences of people who inject drugs while admitted to acute care hospitals and found they were subjected to surveillance, harassment, and neglect, and that these therapeutic contexts became risk environments for marginalized persons [20]. They showed that the enforcement of abstinence only policies (i.e., social structures) combined with unmanaged pain and withdrawal can promote the very behaviours it targets to prevent, including illicit substance use during hospital admission and premature departure from the hospital [20].

Data from a Vancouver study showed that 43.9% of PWUD who had been in hospital reported ever using an illicit drug during their hospitalization [21]. To add to growing literature about drug use in hospitals, in this study we focus on the relational, affective, temporal and fluid elements, as well as acts of resistance associated with it. Unlike other studies that focus on either patient or health care provider perspectives, we explore the accounts of both PWUD and health care providers (i.e., physicians, nurses, pharmacists, social workers, and dieticians) about responses and attempts to manage the use of illicit drugs while admitted to acute care wards in general hospitals.

## Methods

This qualitative, descriptive study is located within a broader program of research about the experience of receiving or providing hospital care to people living with HIV and/or hepatitis C virus (HCV) who use substances [22, 23]. The study was approved by the University of Toronto Human Subjects Review Committee and the St. Michael’s Hospital Research Ethics Board in Toronto.

### Participant recruitment

For the patient population, eligibility criteria included self-reported: 1) HIV and/or HCV infection; 2) current or past use of substances, such as alcohol and drugs in ways that were not clinically indicated, harmful to wellbeing and/or illegal; 3) hospital admission for HIV, HCV and/or drug or alcohol related problems in the past five years; and 4) residing in Toronto or Ottawa, Canada. Patient participants were recruited using poster and electronic advertisements and word of mouth at local AIDS service organizations and community health centres.

Eligibility criteria for health care providers included: 1) currently working in a general hospital in Toronto or Ottawa as a physician, nurse, social worker or other allied health professional; and 2) providing hospital-based health care to this patient group. Physicians on the research team contacted their colleagues at acute care hospitals via personal email and email listservs to provide information about the study and encouraged those interested to contact the study coordinator directly to learn more about the research. With these methods,

### Data collection methods

Patient participants were asked to complete a brief questionnaire and an audio-recorded, face to face, semi-structured interview. The questionnaire included items about socio-demographic characteristics, health status, and substance use. During the semi-structured interview, patient participants were asked about their experiences of accessing acute care in a general hospital, the quality and nature of interactions with hospital-based clinicians, how they negotiated/managed their substance use while admitted to hospital, if and how they used substances while admitted, and what could be done to improve the experience of patients who use substances in acute care settings. Provider participants also completed a brief questionnaire with items about workplace characteristics, work role/position, demographic characteristics, and prior training in addictions and harm reduction. During the audio-recorded, face to face or telephone, semi-structured interview, provider participants were asked about their attitudes and experiences of providing care to this patient population, experiences prescribing opioids, benzodiazepines and psycho-active substances, the management of on-site substance use, procedures for patients leaving against medical advice, any hospital policies pertaining to on-site use of substances that were not prescribed, and recommendations to improve acute care for this population. As compensation, patient participants received $25 CAD cash and return transit fare and provider participants received a $50 CAD gift card for a bookseller.

### Data management and analyses

Audio-recorded interviews, lasting 10–60 minutes, were conducted between 04/2014 and 05/2015 by SR, SCC, AG or CS for patients at the University of Toronto or community organizations where recruitment took place and for providers at the hospital or by telephone. Audio-recordings were transcribed verbatim by a confidential transcriber, verified, corrected, and uploaded to NVivo 10. Using the seven step framework method, team members used line by line coding on several transcripts to identify pre-existing and emergent concepts, collectively discussed codes, developed a code set to be applied to subsequent transcripts, used an iterative approach to re-code transcripts to capture new codes, used Nvivo to capture the codes for each transcript, grouped codes into categories to create the analytic framework, charted the data by transcript and topic (e.g., managing substance use in hospital), and mapped connections between categories to identify and interpret themes in the dataset [24]. Themes were further compared by setting and health provider discipline. To add rigor and ensure the trustworthiness of the interpretation, preliminary analyses were presented to advisory group members, comprised of people living with HIV and/or HCV and who used drugs, for comment and used to refine the interpretation. Questionnaire data were managed using SPSS version 22.0 and descriptive statistics were used to describe the participants.

## Results

For this study, we recruited 24 participants living with HIV and/or HCV who were mostly male, Caucasian and housed; ranged in age from 33–56 years old and reported use of opioids, cocaine and/or many other legal and illegal drugs (see Table 1). We also recruited 26 health care provider participants amongst whom just under half were physicians; more than half were women; their experience working with PWUD varied from 1.6–30 years; and most reported having completed continuing education related to addictions and harm reduction (see Table 2).

**Table 1:** Characteristics of participants (patients) who completed a brief questionnaire and audio-recorded semi-structured interview, April 2014-May 2015.

Patients (n = 24)	% (n)
Gender^a^	
Men	75 (18)
Age (median, range)	49, 33–56
Race	
Caucasian	75 (18)
Indigenous	12.5 (3)
Indigenous and Caucasian	8.3 (2)
Housing Status	
Owned or rented housing	70.8 (17)
Supportive or transitional housing	25.0 (6)
Paid employment in previous 12 months	50 (12)
HIV/HCV Status	
Living with HIV (HCV negative or unknown)	45.8 (11)
Living with HCV (HIV negative)	8.3 (2)
Co-infected	45.8 (11)
Self-reported physical health^b^	
Excellent, very good	37.5 (9)
Good, Fair	54.17 (13)
Poor	8.3 (2)
Self-reported mental health^b^	
Excellent, very good	16.7 (4)
Good, Fair	79.2 (19)
Poor	4.2 (1)
Self-reported substance use ever, past 3 months	
Tobacco	87.5 (21), 75 (18)
Alcohol	95.8 (23), 79.2 (19)
Cannabis	100 (24), 83.3 (20)
Prescription opioids	83.3 (20), 50.0 (12)
Street opioids	62.5 (15), 20.8, (5)
Cocaine	91.7 (22), 62.5 (15)
Stimulants	54.2 (13), 16.67 (4)
Methamphetamine	83.3 (20), 33.3 (8)
Sedatives	75 (18), 37.5 (9)
Hallucinogens	79.2 (19), 0(0)
Clinically significant alcohol use^c^	58.3 (14)
Clinically significant drug use^c^	91.7 (22)
Hospital stays in previous 12 months	
AT least once	79.2 (19)
Three or more	20.8 (5)

Column percents shown. Values in parentheses are numbers of participants. When values were missing from some participants, proportions were calculated among the remainder with non-missing information.

^a^Gender options included male, female and transgender. None identified as transgender.

^b^Participants were asked, ‘In general, would you say your (physical/mental) health is …?’ Options included: Poor, fair, good, very good, excellent.

^c^The CAGE and CAGE-AID Screening Tools were used; participants who answered, ‘yes’ to two or more questions were considered to have clinically significant alcohol or substance use, respectively.

**Table 2:** Characteristics of participants (health care providers) who completed a brief questionnaire and audio-recorded semi-structured interview, April 2014-May 2015.

Providers (n = 26)	% (n)
Gender^a^	
Women	53.8 (14)
Age (median, range)	45, 29–60
Race	
White only	75 (18)
Other (non-white)	26.9 (7)
Type of provider	
Physician	42 (11)
Registered Nurse	19 (5)
Pharmacist	15 (4)
Social Worker	15 (4)
Dietician	3.8 (1)
Nurse Practitioner	3.8 (1)
Work experience (years) with	Average, range
People living with HIV	15.25,1.6–30
People who use drugs/are hazardous drinkers (average, range)	15.72, 1.6–30
Continuing education in any of the following areas^b^	% (n)
Drug addiction	69 (18)
Hazardous drinking	42 (11)
Treatment of substance use disorders	62 (16)
Harm reduction	62 (16)

Column percents shown. Values in parentheses are numbers of participants. When values were missing from some participants, proportions were calculated among the remainder with non-missing information.

^a^Gender options included male, female and transgender. None identified as transgender.

^b^Participants were asked, ‘Since your training, have you attended any continuing education courses, seminars or conferences about …?’ Options included: yes, no.

In this analysis, we trace how lack of institutional policy regarding on premise illicit drug use created an environment where individual clinicians developed informal policies to guide their decisions. These informal policies, based on clincians’ personal beliefs and values, were powerful and for some patients created a risk environment that reproduced stigma and led to hospital admissions characterized by pain and discomfort, early discharge and drug-related risk. To understand what behaviours and situations these informal policies were designed to address, we begin first with a description of what motivates on-premise illicit drug use, what substances are consumed, and how they are consumed.

### The urge to use drugs was sometimes overwhelming

Many patient participants spoke about using psycho-active substances not prescribed to them during a hospital admission: “*If you're a drug addict on the street*, *you're a drug addict in there*. *You know*? *You're not going to stop using ‘cause you're in the hospital*. *(laugh)”* (Ottawa patient-33). Attempts to avoid negative experiences such as withdrawal, boredom, sadness, loneliness and/or pain were commonly cited as reasons for using drugs that had not been prescribed. Exemplifying this are the remarks from Toronto patient-24 who had tried to stay:”*high every day*. *That's dealing (with) boredom and making sure I don't get sick* [experience withdrawal], *because heroin's physically addicting”*. Others spoke to the negative experience of sitting idle in the hospital and “*after*, *… I had*, *sort of caught up with my sleep*, *then it became really boring … loneliness*, *sadness… You just want to not feel like that*. *So*, *it just*, *that urge just is sometimes overwhelming”* (Ottawa patient-39).

Patients described how their self-reported level of pain was not always believed, tolerance to opioids was ignored, and requests for higher doses of pain medications were disregarded and labelled as drug-seeking attempts. Toronto patient-21 recounted: *“It was hell getting…my pain meds*. [Medical staff] *were like ‘You're on forty five milligrams three times a day*? *I find that hard to believe* [that you are still in pain]’”. These experiences discouraged patients from seeking future health care in a timely fashion: *“I'm afraid to go*, *because I don't want to have pain*. . . .*So for me*, *if I knew it was guaranteed that my pain would be looked after in the hospital*, *I would go*. *But otherwise*, *I'm not going to go*.*”* (Toronto patient-27).

Some patients used the drugs they possessed when admitted. Others left the hospital to buy drugs, including Toronto patient-29 who described going out to buy drugs to cope with acute pain:

*“It happens all the time*, *almost every visit that I've been there*, *whether it be me or my partner*, *always going out and getting morphine or what have you*, *to increase the pain medication and so on…*.*I could cite the time my partner was in … for ten hours*, *and in severe pain…I literally went out*, *picked up the morphine*, *came back* [to the emergency department] *and was administering it to her*, *as the nurse opens the curtain*, *'What are you doing*?*' And I'm like*, *'Well*, *you don't want to deal with it*, *so I'm dealing with it*.*' Surprisingly*, *they didn't really do anything about it*.*”*

Participants noted it was easy to obtain their drug of choice given the proximity of the hospital to parks as reflected by Toronto patient-26: “*How did I manage my pain*? …*Walked out of the hospital and right to the park…*. *the drug dealers sit in the park*, *all day*, *all night*, *so* … *you just go out and buy it”*. Patients, including Toronto patient-29, noted that dealers had gotten ‘wise’ to the potential to buy and resell drugs at premium prices: *“You can make a killing in the hospital*. *(laugh)…So*, *there's people that actually do sit out front of the hospital*, *all day long and … hustle people and see if they can buy their meds from them… there's huge money in morphine and that*.*”* Others relied on friends to bring drugs as recounted by Toronto patient-21. *“When you're in isolation*, *they have an alarm on your door… So I couldn't get out*. *So my friends had to bring it in*.*”*

Those who abstained from using drugs while in hospital did so because they faced barriers to using drugs, which included: having been too sick to use substances, lacking money to buy substances, and inability to leave their rooms to buy drugs and/or to have substances delivered to them in hospital. Some attributed their abstinence to personal principles noting ‘it was not right’ to use while in hospital or noted as Toronto patient-27, a stimulant user, *“it's not fun to party when you don't feel good*”.

### Concealing substance use

To avoid hassles, reprimands, and/or discharge, many patients spoke about trying to conceal their substance use from health care providers: “*I’ll just walk in with my sunglasses on*. *They won't even notice*. *I am pretty good at disguising my personality when I'm high*.*”* (Ottawa patient-39). Some patients said that they would *“sneak in the bathroom*. *Not smoking*, *injecting and do it and quickly and where no one can see you*. *lock the door to the bathroom… you don't leave evidence behind*.*”* (Ottawa patient-32). Others described waiting until the evening when fewer staff were present: “*I didn't do it during prime time*. *…The first time I… did use*, *and then the nurse came in… And that's when I realized*, *'There's no way I can use during the day*. *I'm going to get caught*.*'”* (Toronto patient-15). Patients mentioned that it was easier to disguise the use of opioids because stimulants, such as crack cocaine, could lead to more discernible changes such as anxiety, aggression, restlessness, irritability and/or twitching. Others concealed use, not to avoid hassles, but to show respect for others in the hospital: “*I also used some cocaine… after the surgery I had* … *for my fibula*. . . . *I was very discreet*. *I had this room to myself*. . . .*I think it's best not to make a scene*, *not to make your drug use everybody else's problem too*.*”* (Toronto patient-23). Toronto patient-26 described sneaking out to conceal her use “*So*, *I’d wait until nobody was at the front… I’d hear the other people’s bells going off*.” However, this participant also remarked that she did not always conceal her use and that health care providers were aware of her use “*Trust me*, *they know…*. *They’d be standing at the door and I’d do my toke and… be like WHAT*? *(laugh)… They just ignored me for some reason*.” (Toronto patient-26).

When asked, some of the health care providers interviewed said that they were unaware of on-site substance use. A physician said: *“I don't know if I ever saw this* [substance use] *in the hospital” (*Toronto physician-32). Others acknowledged substance use happened during hospitalizations and suggested it was often well concealed by the patients: *“I agree that* [patients use substances], *you know*, *especially for substances that don't cause such an acute cognitive change*, *like*, *you know*, *for example*, *cannabis*. *Like*, *yeah*, *it's very well hidden sometimes*, *yeah*.” (Toronto physician-5).

### A spectrum of informal ‘policy’ responses to illicit drug use

With two exceptions, none of the participants–patient or provider–could identify a hospital policy specific to substance use. For example, a Toronto social worker-11 said: “*I just don't know what our policy is here”* and Toronto patient-27 remarked: ‘*I don’t think there are*. *They have mean security guards*. *That’s about it I think*.*”* Only one of the hospitals where we recruited clinicians had an addiction consulting service (ACS). None of the clinicians interviewed from this hospital noted that they had sought assistance from this group to address illicit drug use. One of our participants was a member of this service and also noted that there was no formal policy in place. In the absence of formal policies, responses to observed or suspected illicit drug use were determined by individual health care providers. Their responses varied from attemptsto enforce abstinence, to efforts to mitigate the risks of illicit drug use, to prescribing medications to manage withdrawal and other symptoms. Toronto physician-5 remarked, “*I've seen a spectrum of different approaches*. *You know*, *one is to call the police*, *right*? *A second is to get some addictions help*. *Third is to cut off their opioids*.*”* The type of response could vary between health care providers, across shifts and as such, patients could experience varied responses across a single admission.

Toronto patient-22, the first exception, remarked when asked about a hospital policy: “*Ah no*. *Oh*. *Yeah*, *well in the psych ward they do…I can’t remember exactly* [what they said] *but they basically read you the riot act*. *They warn you severely*.” Ottawa patient-38 spoke about being told once when he was 14 years old that “*if I was caught in my room either drinking or smoking*, *smoking anything*, *that I would be arrested depending on what was found*, *and have to leave*.*”* For patients, awareness of so-called ‘policies’ only happened after they were caught or suspected of having used an illicit drug. When asked if prior knowledge of ‘policies’ might influence their behaviour, Ottawa patient-34 exemplified the sentiment amongst those who had used while admitted: *“No*, *I would have done it anyway*. *So when I really want to get high*, *I want to get high…it wouldn’t have helped too much*.*”*

### Threats of discharge to enforce abstinence

The strategies used by health care providers to enforce abstinence included: immediate discharge, threats to discharge if drug use continued, increased behavioural monitoring, behavioural contracting to enforce abstinence, confiscating drugs and/or use equipment to prevent future use, and/or reduction or termination of prescription medications. Discharging patients found to be using illicit drugs was justified by personal beliefs that hospital environments should be drug-free and further defended as a measure to reduce risk to staff and other patients. Toronto social worker-11 described how patients would be threatened if they used drugs: *“I certainly know that when a patient hits a certain level of belligerent and difficulty…then the management will step in and … have a more serious talk … infer that they don't need to be in hospital if they're not going to play by the rules… put a bit of the fear in them*.*”* One patient participant, admitted for a broken mandible, was discharged when his illicit drug use was detected: *“Yeah*, *I got my girlfriend to bring me morphine… they kicked me out of the hospital really*, *because of it*.*” (Ottawa patient-33)*.

Lack of institutional policy allowed health care providers to determine if they would discharge a patient following the first detection of substance use or after a warning as exemplified by the remarks by Toronto physician-4 “‘*You know*, *if you are going to smoke crack in the washroom again*, *we're just going to discharge you*, *regardless of how sick you might be*, *because that's not tolerated… It actually puts other people at risk and themselves at risk…if this activity were to persist that it would be grounds for discharge*.*'”*

Increased monitoring of patients and confiscation of drug use equipment were other methods used to discourage illicit drug use: “*patients have…smoked crack or were caught with alcohol…*. *that's just physically removed from them*.*”* (Toronto dietician-31). Ottawa nurse-22 described a gradual escalation of responses to suspected drug use:

*“Obviously*, *I would start by asking the patient*. *And you know*, *sometimes*, *you know that they're lying to you*, *because the signs are there*, *that they're all of a sudden high and they weren't when you went in*, *let's say*, *fifteen minutes earlier*. *So…the doctors are the first to call*. *And then*, *sometimes*, *we've had to have security come and search the room and their belongings*, *unfortunately*, *because they've stashed stuff in secret places in the room*.*”*

About increased monitoring, Toronto patient-16 said “*Everywhere I went*, *I’d turn around… and she’d* [hospital worker] *be there …*. *I just started sneaking around her eventually*.” Ottawa patient-33 noted: *“So they kicked me out* [for using]. *They monitor you a little more closely when you're a drug user*, *right*? *Like*, *they test you more often for …drugs… before they test you for what they're actually looking for*.*”* Ottawa patient-33 and others noted that not only were they monitored more often but also that physicians and nurses “*Just the way they talk to you and that*, *they're a little more stern and that*. *You know*? *There's not*, *there's no*, *like*, *you can't bicker…it turns into an argument*, *but it's like*, *there's no changing their mind*.*”*

Some physicians feared the consequences (e.g., overdose) of prescribing medications to patients who were not honest about their illicit drug use during the admission and cancelled medication orders for pain, anxiety, and/or depression: “*It makes us*, *ah*, *less engaged with them; a lot more careful about*, *you know*, *their privileges off the ward or provision of narcotics or*, *benzos for anxiety …cause of the violation of the agreement or the trust between us*, *absolutely changes*.*”* (Ottawa physician-23). Not all physicians, including Ottawa physician-36, agreed with cancelling prescriptions because they worried that patients would leave against medical advice:

*“Well*, *the flip side… I talk about that story where somebody was not swallowing his oral morphine*. *And he was crushing them in the washroom*. *He had a syringe hidden in the ceiling and then he was injecting himself*. *They found him*. *They make the rules even harder*. *And the guy left*. *Had endocarditis and he left… I wouldn't be surprised if he's dead by now* … *What I dislike about it is instead of facing the situation of 'Okay*, *you have an addiction problem and you need to take morphine and how can we handle that while you're in hospital*?*' It was kind of a conservative approach of 'Oh no*, *you're a criminal and we'll make your life harder’*.*”*

### Prevention of premature departure

In contrast to health care providers who used discharge and increased monitoring to enforce abstinence during hospital admissions, others tried to mitigate the risk of early discharge. Acknowledging the lack of official policies and lack of expertise about how best to help patients, these health care providers prioritized keeping patients in the hospital. Toronto nurse-8 said she would turn: “a *blind eye to things”* and Ottawa physician-16 spoke about allowing patients to go for “*a smoke break*. *They need to get some fresh air*. . . . *you don't usually get in their way*.” This physician also noted that after going outside “*They come back and they're all relaxed and ah*, *and you know*, *they'll often apologize for being a pain in the ass*, *and they're fine until they need their next hit*.*”* (Ottawa physician-16). Ottawa patient-33’s comments were supportive of this approach “*It would be helpful if they accepted the fact that it was going to happen… staff that could adjust to that*.*”* Toronto patient-15 expanded further saying “*I think they should have more of an open approach… Well depending on the behaviour of the person*. *If you are rowdy*, *if they using and it’s not affecting anybody*, *why not let them use*?.*”* Toronto patient-26 also hoped that clinicians would: *“just ignore it… if it doesn’t bother anybody*, *I don’t see what the big problem is”* but this hope often came with a worry that they might receive a lower standard of care: “*And you're a drug user… they forget about your meds…they're later with you or run to the other patients before they run to you*. . . .*”*

Although not commonly discussed, some providers, including Toronto nurse-6, tried to help patients manage their use of drugs: “*If I were to find them using*, *I would probably just say 'You know*, *you really can't do that here*. *The hospital*, *first of all*, *doesn't permit drugs in the hospital that aren't prescribed…Go out*, *do what you have to do and then come back*.*'”* Toronto physician-3 also said he would encourage patients to “*smoke them outside*. *Don't smoke them in the bathroom… it creates a whole bunch of other issues that I can't actually*, *I have limited powers*.”

A small number of physicians believed that efforts to force patients to be abstinent would not lead to long term change for the patient and instead they tried to prevent withdrawal, manage cravings, and/or to manage pain and reduce patient and provider conflict: *“I tend to try and take the easy route and try and provide them what I think they need*, *what they crave*. *otherwise… they leave hospital against medical advice* …*And if that means giving them drugs*, *then so be it*, *you know*?*”* (Ottawa physician-23). Ottawa physician-24 remarked: *“In the cocaine using*, *withdrawal's less of a concern*. *I might calm them down with some Ativan*, *just acutely*. *Usually they just sleep it off*.*”* Ottawa physician-23, although uncomfortable with providing substitution dosing, remarked about a street- involved woman living with HIV and HCV who was admitted with endocarditis:

*“We need to treat her for six weeks*. *We cannot do this safely as an outpatient… let's get her on a dose so that she does not crave it… she was in isolation*. *She was also MRSA positive*. *And got three times daily… MS Contin or something like that…it was six weeks of kind of*, *swallowing what feels wrong*, *that is*, *basically giving an addict what they're addicted to*. *On the other hand*, *it is realistic and we're not going to change the world with a short admission like this*. *And addiction's a little bit more complicated than just denying someone their drugs*, *so*.*”*

Substitution dosing was justified as a measure to ensure consistency of approach across patients, as exemplified by the remarks of Ottawa physician-35: “*If somebody was smoking in the hospital*, *they cannot smoke*. *You give him a patch…Somebody is using morphine in the street and*, *or heroin and he goes in the hospital*, *you give him nothing*. *Right*? *… we should have at least the same standards for smokers and for people using other substances*.*”*

Others hoped that the response might include a conversation about drug treatment and about what might happen next “*It can work*, *one of two ways*. *It can be helpful*, *or I think push the patient away from the clinical aspect*.*”* (Toronto patient-20).

## Discussion

In this study, patient participant accounts of substance use in hospital rooms and bathrooms as well as outside of the facility while admitted align with existing literature and reinforce that common reasons for in-hospital substance use include avoiding physical withdrawal and managing pain during hospital admissions [20, 25]. Patients described obtaining substances from their visitors or leaving the unit to obtain substances outside or from drug dealers on hospital property. Providers highlighted gaps in institutional guidance to appropriately respond to in-hospital substance use. Patients attempted to conceal illicit drug use in environments with no institutional policies about such use leading to varied responses (e.g., reprimands, loss of privileges/medications, discharges) that were inconsistent with the principles of patient centred care and reflected clinicians/providers’ personal beliefs about illicit drug use [26–28]. These responses did not attend to risks such as overdose and proper disposal of drug use equipment that arise from concealed drug use and for the most part did not reflect an attempt to address the physical and emotional comfort of the patient, a hallmark of patient centred care [29].

A contributor to on-premise use is the real and perceived lack of access to appropriate pain management. Fear of pain results in delays seeking medical care, leading to worsened health problems as has been documented here and elsewhere [17, 20]. Previous studies have highlighted the challenges that providers face in prescribing opioids to individuals with substance use disorders, and variation in prescribing practices [30]. Addiction Consulting Services (ACS) may be a model of care that could help to address some of these issues. [31–33]. The models vary but ACS are typically staffed by physicians, nurses, social workers and/or peer workers who work in consultation with attending physicians to provide services such as a comprehensive addiction history, physical examinations, withdrawal management, and initiating pharmacotherapies where indicated [34, 35]. Existing research documents that physicians, nurses and other health care providers often feel ill-equipped to address the complex needs of patients with substance use disorders and ACS have emerged to improve the care and outcomes for inpatients who use drugs. These services have been shown to successfully engage patients in care [34, 35], but estimates suggest that less than ten of these services exist in North America [36]. While an ACS was in operation at one of the hospitals from where we recruited, its existence did not coincide with the development and implementation of a hospital-wide policy regarding illicit drug use. ACSs seem well-placed to also contribute to the development of institutional policies regarding responses to illicit drug use while admitted. However, as we have shown there is great variability in the perspectives of clinicians in how to respond. Utilization of a patient centred approach [27] that incorporates the voices and perspectives of PWUDis crucial to avoid formalization of policies that reproduce a risk environment characterized by stigma, pain and discomfort, early discharge, and drug-related risk.

Past studies have also shown that providers have negative perceptions of PWUD including labelling them as ‘drug-seeking’ or believing patients will sell their prescription opioids, resulting in reluctance to prescribe [30, 37]. While our findings substantiate the suspicion about the sale of prescribed medications within close proximity to the hospital, we only heard accounts of PWUD patients buying in this market, not selling into it. It could be that patients feared disclosing ‘trafficking’ behaviours and did not fully disclose this behaviour. While hospitals might respond by recommending police ‘crack-down’ on this practice within the vicinity of the hospital, this approach may not achieve the intended goal of reducing on-premise drug use. Evidence of crack-downs in community settings by police have been shown to create greater risks for PWUD [38–40]. Better management of pain and withdrawal, and/or initiation of substitution treatment might reduce this illegal market. However, this would depend on improved trust between patients and their healthcare providers, and on improved training for health care providers and/or referral.

Our study contributes to the small, but growing, body of literature and theory about drug use in hospitals and the role of harm reduction by illuminating the relational, affective, temporal, fluid, and ambiguous elements, as well as acts of resistance. First, our study provides both provider and patient perspectives, and highlights relational issues which affect care such as behaviour following use, and affective issues related to fear, anger, anxiety, and disappointment from both patients and providers. The relational is extended into the narratives of patients who supported each other by sharing drugs in hospital with friends and loved ones, and providers who turned a blind eye to these strategies. In addition to the embodied experience of drug use and withdrawal which is well documented in the literature, our findings highlight the temporal elements and how time passes when people are unable to use and the devastating impact of boredom and isolation. Despite the perception of the hospital as fixed, patients moved between the hospital and surrounding community drug using spaces, and dealers moved from those spaces into hospitals, with relative ease making for fluid borders. While much attention has been given to discriminatory and punitive polices in hospitals, we heard about the absence of policy and what this void enables or constrains. Such policy ambiguity empowers some care providers to subject patients to searches and expulsion while it empowers others to provide substitution dosing on a case by case basis. That is, we heard accounts of resistance from both patients and providers. Hospitals are indeed risk environments, but where there is risk there is also opportunity, including for developing client centred care approaches. We invite others to take up these theoretical threads in future research that recognizes the complexity of managing substance use in hospitals. Such an orientation invites thinking about drug use in hospitals as an assemblage of relations, bodies, drugs, affects, temporalities, and policies [41].

While our study illuminates important patient and provider perspectives, there are limitations to consider in its interpretation. Our study findings are based on qualitative data collected about experiences in academic hospitals in two large cities in Canada. Given that participants spoke about experiences that took place in the five years preceding the interviews, their recall of experiences may be influenced by time and other subsequent events. In addition, we recruited people living with HIV and/or HCV and who also use illicit drugs people, a very specific sub-population of people who use drugs whose experiences may not reflect those of PWUD without these infections. Additionally, we recruited people who reported the use of any illicit substance without a prescription and our sample precluded sub-analysis by substance type and/or route of administration. Eligibility for provider participation included delivering care to those living with HIV and/or HCV and this provider group may have different perspectives and attitudes compared to other specialists or general medical providers. While the findings are subject to these limitations and the extent to which the data can be generalized across place and time are not clear, our findings to add to a growing literature that reports similar experiences in other settings suggesting that these experiences are more generalized than specific to person, place or time [20, 25].

There are increasing calls for implementation of harm reduction approaches and interventions (e.g., needle and syringe programs and supervised consumption services) in hospitals [21, 42] but uptake has been slow. Nevertheless, one evaluation of a hospital-based needle and syringe program showed promise for implementation in this setting [43]. Several studies demonstrate that hospital-based harm reduction interventions would be acceptable to patients who use substances, may encourage them to stay to complete their treatment, and minimize drug-related risks [44]. Further, McNeil et al [20] explored the acceptability of incorporating harm reduction interventions into hospital settings with people who inject drugs and found that harm reduction approaches in hospital were necessary to promote patient centred care. They suggested that harm reduction approaches could improve patient-provider relationships and reduce discharge against medical advice [27]. However, implementing harm reduction on its own will not address the issues of mistrust and the past traumatic experiences of PWUD in healthcare systems (e.g., being mistreated and cut-off of pain medication), and lack of patient centred care approaches. To help address this gap we call for trauma-informed care and policies attuned to the health needs and lived realities of PWUD [45–48]. Such an approach is attuned to power in general, but further considers issues of cultural safety for Indigenous persons who have been historically marginalized in healthcare [49, 50]. As many of our participants noted, imposing abstinence during a hospitalization is likely to lead to delayed care, discharge, and further mistrust and the potential for increased mortality. A spectrum of harm reduction, from facilitating safer use to substitution therapy may promote greater retention in care, and create opportunities for discussions about drug treatment on patients’ terms.

## References

[1] Roxburgh A, Hall WD, Dobbins T, Gisev N, Burns L, Pearson S, et al Trends in heroin and pharmaceutical opioid overdose deaths in Australia. Drug and Alcohol Dependence. 2017;179:291–8. 10.1016/j.drugalcdep.2017.07.018 28826104

[2] European Monitoring Centre for Drugs and Drug Addiction. Drug-related deaths and mortality in Europe. Luxembourg: Publications Office of the European Union; 2019.

[3] Australian Bureau of Statistics. 3303.0–Causes of Death, Australia, 2018. ABS Canberra (AUST); 2019.

[4] Kalkman GA, Kramers C, van Dongen RT, van den Brink W, Schellekens A. Trends in use and misuse of opioids in the Netherlands: a retrospective, multi-source database study. The Lancet Public Health. 2019;4(10):e498–e505. 10.1016/S2468-2667(19)30128-8 31444001

[5] Kochanek KD, Murphy SL, Xu J, Arias E. Mortality in the United States, 2016 NCHS Data Brief, no 293. Hyattsville (MD): National Center for Health Statistics,; 2017. 29319473

[6] Orpana Heather M, Lang Justin J, George Diana HJ. At-a-glance-The impact of poisoning-related mortality on life expectancy at birth in Canada, 2000 to 2016. Health promotion and chronic disease prevention in Canada: research, policy and practice. 2019;39(2):56. 10.24095/hpcdp.39.2.03 PMC6394823 30767855

[7] National Report: Apparent Opioid-related Deaths in Canada [Internet]. Government of Canada. 2019 [cited May 28, 2019]. Available from: https://health-infobase.canada.ca/datalab/national-surveillance-opioid-mortality.html.

[8] Rudasill SE, Sanaiha Y, Mardock AL, Khoury H, Xing H, Antonios JW, et al Clinical Outcomes of Infective Endocarditis in Injection Drug Users. Journal of the American College of Cardiology. 2019;73(5):559–70. 10.1016/j.jacc.2018.10.082 30732709

[9] Canadian Institute for Health Information. Opioid-Related Harms in Canada. Ottawa, ON: CIHI; 2018 12.

[10] Velez CM, Nicolaidis C, Korthuis PT, Englander H. “It’s been an Experience, a Life Learning Experience”: A Qualitative Study of Hospitalized Patients with Substance Use Disorders. Journal of General Internal Medicine. 2017;32(3):296–303. 10.1007/s11606-016-3919-4 27957661 PMC5331007

[11] Priest KC, McCarty D. Role of the Hospital in the 21st Century Opioid Overdose Epidemic: The Addiction Medicine Consult Service. Journal of Addiction Medicine. 2019;13(2):104–12. 10.1097/ADM.0000000000000496 30608266 PMC6417955

[12] Day C, Ross J, Dolan K. Hepatitis C-related discrimination among heroin users in Sydney: drug user or hepatitis C discrimination? Drug and Alcohol Review. 2003;22(3):317–21. 10.1080/0959523031000154463 15385226

[13] Haber PS, Demirkol A, Lange K, Murnion B. Management of injecting drug users admitted to hospital. The Lancet. 2009;374(9697):1284–93. 10.1016/S0140-6736(09)61036-9 19819393

[14] Miller NS, Sheppard LM, Colenda CC, Magen J. Why physicians are unprepared to treat patients who have alcohol-and drug-related disorders. Academic Medicine. 2001;76(5):410–8. 10.1097/00001888-200105000-00007 11346513

[15] Rosenthal ES, Karchmer AW, Theisen-Toupal J, Castillo RA, Rowley CF. Suboptimal addiction interventions for patients hospitalized with injection drug use-associated infective endocarditis. The American journal of medicine. 2016;129(5):481–5. 10.1016/j.amjmed.2015.09.024 26597670

[16] Merrill JO, Rhodes LA, Deyo RA, Marlatt GA, Bradley KA. Mutual mistrust in the medical care of drug users: the keys to the "narc" cabinet. J Gen Intern Med. 2002;17(5):327–33. 10.1046/j.1525-1497.2002.10625.x 12047728 PMC1495051

[17] Chan Carusone S, Guta A, Robinson S, Tan DH, Cooper C, O’Leary B, et al “Maybe if I stop the drugs, then maybe they’d care?”—hospital care experiences of people who use drugs. Harm Reduction Journal. 2019;16(1):16 10.1186/s12954-019-0285-7 30760261 PMC6373073

[18] Pecoraro A, Royer-Malvestuto C, Rosenwasser B, Moore K, Howell A, Ma M, et al Factors contributing to dropping out from and returning to HIV treatment in an inner city primary care HIV clinic in the United States. AIDS Care. 2013;25(11):1399–406. 10.1080/09540121.2013.772273 23428205

[19] Rhodes T. The ‘risk environment’: a framework for understanding and reducing drug-related harm. International journal of drug policy. 2002;13(2):85–94.

[20] McNeil R, Small W, Wood E, Kerr T. Hospitals as a 'risk environment': an ethno-epidemiological study of voluntary and involuntary discharge from hospital against medical advice among people who inject drugs. Soc Sci Med. 2014;105:59–66. 10.1016/j.socscimed.2014.01.010 24508718 PMC3951660

[21] Grewal HK, Ti L, Hayashi K, Dobrer S, Wood E, Kerr T. Illicit drug use in acute care settings. Drug and alcohol review. 2015;34(5):499–502. 10.1111/dar.12270 25944526 PMC4636467

[22] Strike C, Guta A, de Prinse K, Switzer S, Chan Carusone S. Living with addiction: the perspectives of drug using and non-using individuals about sharing space in a hospital setting. Int J Drug Policy. 2014;25(3):640–9. 10.1016/j.drugpo.2014.02.012 24679487

[23] Switzer S, Guta A, de Prinse K, Chan Carusone S, Strike C. Visualizing harm reduction: Methodological and ethical considerations. Soc Sci Med. 2015;133:77–84. 10.1016/j.socscimed.2015.03.040 25841098

[24] Gale NK, Heath G, Cameron E, Rashid S, Redwood S. Using the framework method for the analysis of qualitative data in multi-disciplinary health research. BMC Med Res Methodol. 2013;13:117 10.1186/1471-2288-13-117 24047204 PMC3848812

[25] Grewal HK, Ti L, Hayashi K, Dobrer S, Wood E, Kerr T. Illicit drug use in acute care settings. Drug Alcohol Rev. 2015;34(5):499–502. 10.1111/dar.12270 25944526 PMC4636467

[26] Kolind T, Hesse M. Patient‐centred care—perhaps the future of substance abuse treatment. Addiction. 2017;112(3):465–6. 10.1111/add.13673 28168786

[27] McNeil R, Kerr T, Pauly B, Wood E, Small W. Advancing patient-centered care for structurally vulnerable drug-using populations: a qualitative study of the perspectives of people who use drugs regarding the potential integration of harm reduction interventions into hospitals. Addiction. 2016;111(4):685–94. 10.1111/add.13214 26498577 PMC4801725

[28] Stewart M. Towards a global definition of patient centred care: the patient should be the judge of patient centred care. British Medical Journal Publishing Group; 2001.

[29] Kitson A, Marshall A, Bassett K, Zeitz K. What are the core elements of patient‐centred care? A narrative review and synthesis of the literature from health policy, medicine and nursing. Journal of advanced nursing. 2013;69(1):4–15. 10.1111/j.1365-2648.2012.06064.x 22709336

[30] Berg KM, Arnsten JH, Sacajiu G, Karasz A. Providers' experiences treating chronic pain among opioid-dependent drug users. J Gen Intern Med. 2009;24(4):482–8. 10.1007/s11606-009-0908-x 19189194 PMC2659151

[31] Wakeman SE, Metlay JP, Chang Y, Herman GE, Rigotti NA. Inpatient addiction consultation for hospitalized patients increases post-discharge abstinence and reduces addiction severity. Journal of general internal medicine. 2017;32(8):909–16. 10.1007/s11606-017-4077-z 28526932 PMC5515798

[32] Englander H, Weimer M, Solotaroff R, Nicolaidis C, Chan B, Velez C, et al Planning and designing the improving addiction care team (IMPACT) for hospitalized adults with substance use disorder. Journal of hospital medicine. 2017;12(5):339 10.12788/jhm.2736 28459904 PMC5542562

[33] Murphy MK, Chabon B, Delgado A, Newville H, Nicolson SE. Development of a substance abuse consultation and referral service in an academic medical center: challenges, achievements and dissemination. Journal of clinical psychology in medical settings. 2009;16(1):77–86. 10.1007/s10880-009-9149-8 19219627

[34] Wakeman SE, Pham-Kanter G, Donelan K. Attitudes, practices, and preparedness to care for patients with substance use disorder: results from a survey of general internists. Substance abuse. 2016;37(4):635–41. 10.1080/08897077.2016.1187240 27164025

[35] Monks R, Topping A, Newell R. The dissonant care management of illicit drug users in medical wards, the views of nurses and patients: a grounded theory study. Journal of advanced nursing. 2013;69(4):935–46. 10.1111/j.1365-2648.2012.06088.x 22776007

[36] Weinstein ZM, Wakeman SE, Nolan S. Inpatient addiction consult service: expertise for hospitalized patients with complex addiction problems. Medical Clinics of North America. 2018;102(4):587–601. 10.1016/j.mcna.2018.03.001 29933817 PMC6750950

[37] Baldacchino A, Gilchrist G, Fleming R, Bannister J. Guilty until proven innocent: a qualitative study of the management of chronic non-cancer pain among patients with a history of substance abuse. Addict Behav. 2010;35(3):270–2. 10.1016/j.addbeh.2009.10.008 19897313

[38] Aitken C, Moore D, Higgs P, Kelsall J, Kerger M. The impact of a police crackdown on a street drug scene: evidence from the street. International journal of drug policy. 2002;13(3):193–202.

[39] Werb D, Wood E, Small W, Strathdee S, Li K, Montaner J, et al Effects of police confiscation of illicit drugs and syringes among injection drug users in Vancouver. International Journal of Drug Policy. 2008;19(4):332–8. 10.1016/j.drugpo.2007.08.004 17900888 PMC2529170

[40] Wood E, Spittal PM, Small W, Kerr T, Li K, Hogg RS, et al Displacement of Canada's largest public illicit drug market in response to a police crackdown. Cmaj. 2004;170(10):1551–6. 10.1503/cmaj.1031928 15136548 PMC400719

[41] Duff C. Assemblages of health: Deleuze's empiricism and the ethology of life: Springer; 2014.

[42] Sharma M, Lamba W, Cauderella A, Guimond TH, Bayoumi AM. Harm reduction in hospitals. Harm reduction journal. 2017;14(1):32 10.1186/s12954-017-0163-0 28583121 PMC5460456

[43] Miskovic M, Chan Carusone S, Guta A, O’Leary B, dePrinse K, Strike C. Distribution of Harm Reduction Kits in a Specialty HIV Hospital. American journal of public health. 2018;108(10):1363–5. 10.2105/AJPH.2018.304600 PMC6137793 30138074

[44] Ti L, Buxton J, Harrison S, Dobrer S, Montaner J, Wood E, et al Willingness to access an in-hospital supervised injection facility among hospitalized people who use illicit drugs. J Hosp Med. 2015;10(5):301–6. 10.1002/jhm.2344 25754871 PMC4412787

[45] Kirst M, Aery A, Matheson FI, Stergiopoulos V. Provider and Consumer Perceptions of Trauma Informed Practices and Services for Substance Use and Mental Health Problems. International Journal of Mental Health and Addiction. 2017;15(3):514–28.

[46] Reeves E. A Synthesis of the Literature on Trauma-Informed Care. Issues in Mental Health Nursing. 2015;36(9):698–709. 10.3109/01612840.2015.1025319 26440873

[47] Raja S, Hasnain M, Hoersch M, Gove-Yin S, Rajagopalan C. Trauma informed care in medicine. Family & community health. 2015;38(3):216–26. 26017000 10.1097/FCH.0000000000000071

[48] Bowen EA, Murshid NS. Trauma-Informed Social Policy: A Conceptual Framework for Policy Analysis and Advocacy. American Journal of Public Health. 2016;106(2):223–9. 10.2105/AJPH.2015.302970 26691122 PMC4815621

[49] McCall J, Pauly B. Sowing a Seed of Safety: Providing Culturally Safe Care in Acute Care Settings for People who use Drugs. Journal of Mental Health and Addiction Nursing. 2019;3(1):e1–e7.

[50] Pauly BB, McCall J, Browne AJ, Parker J, Mollison A. Toward cultural safety. Advances in Nursing Science. 2015;38(2):121–35. 10.1097/ANS.0000000000000070 25932819

